# Impact of an 8-week high-intensity bodyweight interval training on body composition and blood lipid metabolism in young women with overweight

**DOI:** 10.3389/fpubh.2025.1578569

**Published:** 2025-10-17

**Authors:** Emre Yamaner, Tuna Turğut, Ayşe Aksoy, Burhan Demirkıran, Mine Akkuş Uçar, Burhan Başoğlu, Furkan Çamiçi, Muhammed Said Yanar, Alpay Bülbül, Ahmet Ferdi Koç, Tülay Ceylan, Levent Ceylan, Hamza Küçük

**Affiliations:** ^1^Sungurlu Vocational School of Higher Education, Hitit University, Çorum, Türkiye; ^2^Faculty of Sports Sciences, Bartın University, Bartın, Türkiye; ^3^Faculty of Sports Science, Akdeniz University, Antalya, Türkiye; ^4^School of Physical Education and Sports, Mardin Artuklu University, Mardin, Türkiye; ^5^Faculty of Sports Science, Nevşehir Hacı Bektaş Veli University, Nevşehir, Türkiye; ^6^Faculty of Sport Sciences, Hitit University, Çorum, Türkiye; ^7^School of Physical Education and Sports, Osmaniye Korkut Ata University, Osmaniye, Türkiye; ^8^Faculty of Sport Sciences, Aydın Adnan Menderes University, Aydın, Türkiye; ^9^Institute of Graduate Studies, Ondokuz Mayıs University, Samsun, Türkiye; ^10^Yaşar Doğu Faculty of Sport Sciences, Ondokuz Mayıs University, Samsun, Türkiye

**Keywords:** high-intensity interval resistance training (HIIRT), young women, body composition, lipid metabolism, overweight

## Abstract

**Background:**

A physically inactive lifestyle is associated with an increased risk of obesity, dyslipidemia and metabolic disorders, especially in women. While high-intensity training methods have been extensively studied in male populations, the physiological and metabolic effects of high-intensity interval resistance training (HIIRT) in overweight women are understudied. The aim of this study was to investigate the effects of an 8-week HIIRT program on body composition and lipid metabolism in women.

**Methods:**

A total of 30 women (mean age: 23.13 ± 4.03 years, mean BMI: 31.21 ± 2.92 kg/m^2^) participated in an 8-week HIIRT program. Body composition was measured before and after the intervention using bioelectrical impedance analysis (BIA), while blood lipid parameters (triglycerides, total cholesterol, LDL, and HDL) were analyzed. Paired-sample *t*-tests were performed to determine the significance of the changes in body composition and lipid metabolism, using Cohen’s *d* effect size for interpretation.

**Results:**

The HIIRT program led to significant improvements in body composition, with body weight decreasing by 11.4 kg (*p* = 0.001, *d* = 0.96) and fat percentage decreasing by 3.1% (*p* = 0.001, *d* = 0.92). In addition, blood lipid profiles improved significantly, with triglycerides (−8.9 mg/dL, *p* = 0.001, *d* = 0.81, medium effect), total cholesterol (−19.7 mg/dL, *p* = 0,020, *d* = 1.98, large effect), and LDL (−8.2 mg/dL, *p* = 0.004, *d* = 1.96, large effect) decreased, while HDL increased by +10 mg/dL (*p* = 0.006, *d* ≥ 2.0, very large effect). These results underline the positive effects of HIIRT on lipid metabolism and cardiovascular health.

**Conclusion:**

The results suggest that HIIRT is an effective and time-efficient training model for improving body composition and metabolic health in women. The combination of high-intensity interval training principles with resistance exercise optimizes fat oxidation, improves fat metabolism and supports cardiovascular function. Given its efficacy, HIIRT could be a valuable strategy for treating metabolic disorders and reducing the risks of physically inactive behavior. Future research should focus on long-term adaptations and individual variability in metabolic responses to optimize HIIRT programs for broader populations.

## 1 Introduction

A physically inactive lifestyle characterized by prolonged physical inactivity is a major risk factor for several metabolic and cardiovascular diseases, including obesity, dyslipidemia and type 2 diabetes. This lifestyle negatively affects physiological functions by reducing cardiovascular efficiency, impairing metabolic health and increasing the risk of chronic diseases. Therefore, structured exercise interventions such as high-intensity interval resistance training (HIIRT) have gained attention due to their potential benefits in combating these negative effects, especially in physically inactive populations (1).

High-intensity interval resistance training (HIIRT) is a variant of HIIT that specifically integrates resistance-based exercises, typically performed with bodyweight or external loads, within the interval structure of high-intensity bouts and recovery periods. While traditional HIIT primarily focuses on improving aerobic and anaerobic capacity through cardiovascular-based activities (e.g., cycling, running, sprinting), HIIRT emphasizes muscular strength, endurance, and hypertrophy by incorporating exercises such as squats, lunges, push-ups, or resistance circuit movements. As such, HIIRT engages both the muscular and cardiovascular systems simultaneously, promoting greater muscle activation and improvements in both neuromuscular and metabolic adaptations. This distinct training focus makes HIIRT particularly suitable for enhancing body composition and fat metabolism, offering complementary benefits beyond the aerobic improvements typically observed with traditional HIIT (2, 3). According to the American College of Sports Medicine’s Worldwide Survey of Fitness Trends, HIIT was recognized as the top fitness trend between 2014 and 2018 (4, 5). In particular, its role in relation to body composition and health/fitness has been emphasized. In addition to its worldwide (6), regional (7), and national (8) popularity, the effectiveness of resistance-based interval training has been widely used among individuals seeking to improve cardiometabolic health (2, 9, 10). Furthermore, as recently reported, the role of muscle strengthening in metabolic health (11) and the role of bodyweight exercises in energy expenditure among young people has also been emphasized (12).

Despite the growing body of research on high-intensity interval resistance training (HIIRT), most studies have primarily focused on male participants, leaving a significant gap in understanding its effects on women. Physiological differences between men and women, including variations in hormonal regulation, fat metabolism, and muscle fiber composition, may lead to distinct responses to HIIRT (13–16). For instance, estrogen has been shown to influence lipid metabolism and recovery kinetics, suggesting that women may experience unique adaptations compared to men. Furthermore, women generally have a higher proportion of type I muscle fibers and greater reliance on fat oxidation during exercise, which could affect their metabolic and performance outcomes in response to HIIRT. Despite these differences, limited research has specifically examined the impact of HIIRT on sedentary women, making it crucial to investigate whether this training method can be an effective and time-efficient strategy for improving body composition and metabolic health in this population (17). Addressing this gap will contribute to the development of gender-specific exercise prescriptions and provide valuable insights into optimizing HIIRT for women.

The importance of HIIRT programs for sedentary women stems from several key factors. First, this type of training provides an efficient and accessible method for improving physical health in individuals with low levels of physical activity. By combining high-intensity efforts with rest periods, HIIRT allows for cardiovascular conditioning and the development of muscular strength while maintaining metabolic efficiency. Secondly, HIIRT has been associated with improved fat metabolism, fat oxidation and body composition, making it a potential strategy to reduce the risk of obesity and metabolic syndrome. In addition, HIIRT can improve exercise participation and motivation due to its time efficiency, thus removing common barriers to physical activity (18, 19).

Despite the growing body of research demonstrating the effectiveness of HIIT, most studies have focused primarily on male participants, leaving a gap in understanding the effects on women. Given the physiological differences in metabolic and cardiovascular responses between the sexes, investigating the effects of HIIRT in physically inactive women is crucial for the development of targeted training protocols. Furthermore, the combination of high-intensity interval principles with resistance training remains under-researched in terms of their acute and chronic physiological adaptations.

Against this background, the present study aims to investigate the physiological and metabolic adaptations resulting from an 8-week HIIRT program in women, with a particular focus on lipid metabolism and body composition. The study will explore both short-term (acute) and long-term (chronic) physiological responses to assess the efficacy of HIIRT as a resistance training method for improving metabolic health in physically inactive individuals. Specifically, it is hypothesized that the HIIRT program will lead to a reduction in body fat percentage and body weight, contributing to overall improvements in body composition. Furthermore, it is expected that HIIRT will induce favorable changes in lipid metabolism, specifically by reducing LDL (low-density lipoprotein) and triglyceride levels while increasing HDL (high-density lipoprotein) levels. These anticipated adaptations align with previous research suggesting that high-intensity resistance training enhances fat oxidation and improves cardiovascular health markers. By addressing these hypotheses, this study seeks to provide valuable insights for sports science, exercise physiology, and public health, particularly in optimizing resistance training interventions for inactive populations.

## 2 Materials and methods

### 2.1 Research model

The study uses an experimental research design with a pre-test-post-test training group to investigate the physiological and metabolic adaptations induced by 8 weeks of high-intensity interval resistance training (HIIRT) in women. This model was chosen to assess both acute and chronic physiological changes and to provide a structured and controlled assessment of training-induced adaptations. By using a pre-and posttest comparison, the study aimed to identify significant changes in fat metabolism, body composition and other physiological parameters while minimizing confounding variables (20). Participants were recruited through university announcements and social media platforms. After an initial health screening and informed consent process, eligible participants were enrolled into the HIIRT intervention group.

A total of 30 sedentary women aged 18–30 years (mean age: 23.13 ± 4.03 years, mean BMI: 31.21 ± 2.92 kg/m^2^) voluntarily participated in this study. Inclusion criteria were: (1) being classified as overweight or obese (BMI ≥ 25 kg/m^2^), (2) not engaging in regular physical activity (less than 150 min of moderate exercise per week), and (3) having no known cardiovascular, metabolic, or musculoskeletal disorders. Also participants’ physical activity level prior to enrollment was assessed through a structured interview during screening. Participants reported their average weekly frequency and duration of moderate to vigorous physical activity. Those reporting less than 150 min per week were classified as sedentary and eligible for the study (1).

Exclusion criteria included: (1) pregnancy, (2) recent participation in a structured exercise program within the last 6 months, (3) any diagnosed chronic disease affecting metabolic health, and (4) the use of lipid-lowering medications or dietary supplements that could influence lipid metabolism. The study protocol was approved by the Osmaniye University Ethics Committee (Decision No: 35).

### 2.2 Participant selection and study design

A total of 54 women initially responded to the recruitment announcements. After health screening and eligibility assessment, 34 women met the inclusion criteria and were invited to participate. Of these, four women declined participation after learning about the time commitment required for the intervention. Consequently, 30 women were enrolled in the study. No participants dropped out during the 8-week HIIRT program.

To ensure the study was adequately powered to detect meaningful effects, a preliminary power analysis was conducted using G*Power 3.1 software. A one-tailed paired-sample *t*-test was selected with an effect size (*dz*) of 0.80 (large), *α* = 0.05, and desired power (1–*β*) = 0.80. The analysis indicated that a minimum of 18 participants would be required. With 30 participants included in the study, our sample exceeded this requirement, ensuring sufficient power to detect significant within-group effects.

Although HIIRT provides notable metabolic and cardiovascular benefits, it may not be suitable for all individuals, particularly overweight and obese women with specific health conditions. Contraindications include uncontrolled hypertension, severe cardiovascular disease, advanced osteoarthritis, or musculoskeletal limitations (21, 22). Therefore, in this study, all participants underwent health screening, and those with cardiovascular, metabolic, or musculoskeletal disorders were excluded. Informed consent was obtained after participants were fully briefed on the study aims, procedures, and potential risks. All training sessions were supervised by qualified professionals to ensure safety and proper execution. No adverse events were reported during the 8-week intervention. Participants were asked to maintain their usual dietary habits throughout the study and were reminded weekly to avoid making intentional dietary changes. Dietary intake was not formally recorded but participants confirmed adherence through weekly check-ins.

### 2.3 Data collection

#### 2.3.1 Body weight and height measurements

The participants’ body weight and height were measured using a calibrated measuring device with an accuracy of 0.01 kg in kilograms and centimeters. During the measurements, participants were instructed to adopt an upright posture, place their feet flat on the floor, keep their knees straight and heels together and stand upright. They were also asked to step on the scale barefoot to minimize measurement inconsistencies. All body weight and height measurements were conducted in the morning, before breakfast, and at the same time of day for both pre-and post-intervention assessments to ensure consistency.

#### 2.3.2 Bioelectrical impedance analysis (BIA)

Bioelectrical impedance analysis (BIA) has been used since the 1990s as a non-invasive and rapid method for assessing body composition. The BIA measurements in this study were carried out using TANITA brand devices. To ensure the accuracy of the measurements, participants were instructed not to exercise, consume caffeine, or eat breakfast before the measurement. The measurements were taken early in the morning using the Tanita BC 418 model. Before each measurement, the electrodes were cleaned and participants were instructed to walk barefoot on the device to ensure consistent readings.

### 2.4 Biochemical analyses

Blood samples were collected from all participants at baseline (pre-intervention) and after the 8-week HIIRT program (post-intervention) under standardized conditions. To minimize potential diurnal variations, blood collection was performed between 07:00 and 09:00 a.m. following an overnight fast of at least 10 h. Venous blood samples (5 mL) were drawn from the antecubital vein by a trained phlebotomist under sterile conditions.

The collected samples were immediately stored in vacutainer tubes containing EDTA (for plasma separation) and clot activator tubes (for serum analysis). Plasma and serum were separated by centrifugation at 3,000 rpm for 10 min at 4 °C and stored at −80 °C until analysis.

Lipid profile parameters, including triglycerides (TG), total cholesterol (TC), low-density lipoprotein (LDL), and high-density lipoprotein (HDL), were analyzed using an automated biochemistry analyzer (Beckman Coulter AU5800, Brea, CA, USA). The enzymatic colorimetric method was applied for TC and TG quantification, while HDL and LDL levels were determined using homogeneous direct assays. All biochemical analyses were conducted in a certified clinical laboratory by trained personnel following standard operating procedures.

### 2.5 Exercise intervention

The training program consisted of 8 weeks of high-intensity interval resistance training (HIIRT) specifically tailored for women aged 18–30 years. The program included bodyweight exercises to improve muscular endurance, strength and metabolic function while minimizing the risk of injury. The training sessions were performed three times a week (Tuesday–Thursday–Saturday) with moderate to high intensity and a progressive loading approach. Progressive overload was implemented by increasing work interval duration, reducing rest intervals, and adding exercise variations with higher difficulty levels across the 8-week program. Participants were required to attend at least 80% of the scheduled sessions (minimum 20 out of 24 sessions) to be considered compliant with the intervention protocol. If a session was missed due to illness or personal reasons, make-up sessions were offered when feasible. In this study, no participants missed more than two sessions, and all met the attendance criteria.

Participants completed dynamic warm-up exercises before each session and cool-down exercises after training to improve flexibility and recovery.

Training principles:

Warm-up (5–10 min): Light endurance training (marching on the spot, arm circles) + flexibility exercises.
Main workout: The duration of the main workout ranged from approximately 15–25 min per session, depending on the training phase and progressive adjustments in volume and intensity.
Cool down (5–10 min): Static stretching and breathing exercises to promote recovery.

The revised HIIRT program has been designed to be safe, progressive and engaging for women, focusing on full body muscle activation, taking into account the fitness level of participants. Based on individual feedback and physiological responses, adjustments were made to optimize adherence and minimize fatigue (Tables 1, 2) (3).

**Table 1:** High-intensity interval resistance training (HIIRT) program.

Weeks	Intensity	Work interval	Rest interval	Repetitions (sets × rounds)	Method
Week 1–2	60–70% HRR (heart rate reserve)	20 s load	15 s active recovery	2 × 6	Strength-based bodyweight progressive loading
Week 3–4	70–80% HRR	25 s load	15 s active recovery	3 × 6	Strength-based bodyweight progressive loading
Week 5–6	80–90% HRR	30 s load	15 s active recovery	3 × 8	Strength-based bodyweight progressive loading
Week 7–8	85–100% HRR	30 s load	10 s active recovery	4 × 8	Strength-based bodyweight progressive loading

**Table 2:** High-intensity interval resistance training (HIIRT) program.

Day	Exercise 1	Exercise 2	Exercise 3	Exercise 4
Tuesday	Bodyweight squats	Incline push-ups	Glute bridges	Seated leg raises
Thursday	Jumping jacks	Step-ups	Wall sit	Plank shoulder taps
Saturday	Knee-ups	Mountain climbers	Russian twists	Modified burpees

### 2.6 Determining exercise intensity

To ensure the appropriate intensity of the 8-week High-Intensity Interval Resistance Training (HIIRT) program, the training load was individually adjusted using the Karvonen formula, which calculates the target heart rate (THR) based on each participant’s resting heart rate (RHR) and maximum heart rate (MHR). This method allows for a personalized intensity adjustment that considers individual cardiovascular fitness levels (23):

Maximum Heart Rate(MHR)=220−Age

$$\text{Target Heart Rate}\;(\mathrm{THR})=(\begin{array}{l} \%\text{Exercise Intensity}\times \\ \left(\mathrm{MHR}-\mathrm{RHR}\right) \end{array})+\mathrm{RHR}\;$$

Participants’ resting heart rates were measured in a seated position after 5 min of rest before the start of the intervention. To ensure that participants maintained the prescribed intensity during training, real-time heart rate monitoring was conducted using Polar H10 heart rate monitors (Polar Electro, Finland), which provide continuous heart rate tracking with Bluetooth connectivity to mobile devices. Coaches closely monitored heart rate data through the Polar Beat mobile application, ensuring that participants remained within the prescribed heart rate reserve (HRR) range throughout each session.

Participants were encouraged to adjust their effort based on real-time heart rate feedback, and any deviations from the target intensity range were corrected immediately by modifying the pace or workload. This continuous monitoring approach ensured adherence to the planned exercise intensity and minimized the risk of overexertion.

### 2.7 Statistical analysis

Descriptive statistics, including mean and standard deviation, the normality of the difference scores was tested using the Shapiro–Wilk test. The data met the assumptions for parametric testing, and therefore paired-samples *t*-tests were used. Cohen’s *d* effect sizes were calculated as the difference between the pretest and posttest means divided by the pooled standard deviation. Interpretation of Cohen’s *d* followed the extended guidelines proposed by Sawilowsky (24): <0.20 = trivial, 0.20–0.49 = small, 0.50–0.79 = medium, 0.80–1.19 = large, 1.20–1.99 = very large, ≥ 2.0 = huge. The significance level was set at 0.05, and analyses were conducted using SPSS 26.0 software for Windows.

## 3 Results

The mean age of the women in the study group was 23.13 ± 4.03 years, mean height was 163.83 ± 4.77, mean weight was 83.85 ± 13.96 and mean body mass index was 31.21 ± 2.92 (Table 3).

The HIIRT program resulted in a significant reduction in participants’ body weight (−11.4 kg, *p* = 0.001, *d* = 0.96), indicating a large effect size. This suggests a meaningful improvement in metabolic health and a lower risk of obesity-related conditions. Similarly, body fat percentage decreased by 3.1% (*p* = 0.001, *d* = 0.92), reflecting a moderate effect that supports enhanced body composition and fitness.

In terms of blood lipid values, triglyceride levels significantly decreased by 8.9 mg/dL (*p* = 0.001, *d* = 0.81), which represents a moderate effect and indicates improved lipid metabolism. Total cholesterol levels dropped by 19.7 mg/dL (*p* = 0.020, *d* = 1.98), and LDL cholesterol decreased by 8.2 mg/dL (*p* = 0.004, *d* = 1.96), both indicating large effect sizes and substantial improvements in cardiovascular risk markers. Meanwhile, HDL cholesterol increased by 10 mg/dL (*p* = 0.006, *d* ≥ 2.0), showing a very large effect, which is beneficial for heart health. These combined improvements confirm the efficacy of the HIIRT program in positively altering lipid profiles (Table 5).

**Table 3:** Table of participants’ physical characteristics.

Variables	x̄ ± SD
Age (year)	23.13 ± 4.03
Weight (kg)	83.85 ± 13.96
Height (cm)	163.83 ± 4.77
BMI (kg/m^2^)	31.21 ± 2.92

## 4 Discussion

This study was conducted to investigate the effects of 8 weeks of high-intensity interval resistance training (HIIRT) on body composition and fat metabolism in women. A physically inactive lifestyle is associated with various metabolic disorders such as obesity, dyslipidemia and cardiovascular disease, and it is of great public health importance to encourage this population to be physically active. By combining resistance and high-intensity interval training components, HIIRT aims to achieve effective physiological adaptations in less time than traditional training methods.

The study results show that the HIIRT program improved body composition by reducing body weight and body fat (Table 4) and produced positive changes in the blood lipid profile (Table 5). In particular, the significant reduction in triglyceride (TG), total cholesterol and LDL levels and the increase in HDL levels demonstrate the positive effects of HIIRT on cardiovascular health. These results support the applicability of HIIRT as a time-efficient and effective training method for women and provide important contributions to studies in this field. Moreover, the present findings align with existing evidence that increased physical activity is associated with improvements in metabolic profile, including favorable changes in lipid metabolism and body composition (1, 18). These relationships further underscore the importance of promoting regular physical activity in sedentary populations.

**Table 4:** Changes in weight, BMI before and after 8-weeks HIIRT workout program.

Variables	Pre	Post	Change (*Δ*)	*p*	95% CI	Cohen *d*	Effect size interpretation
	x̄ ± SD	x̄ ± SD					
Body weight (kg)	83.8 ± 13.9	72.4 ± 9.3	−11.4 kg	0.001	−15.98 to −6.82	0.96 (medium)	Moderate effect: Indicates a significant reduction in body weight, which may contribute to improved metabolic health and decreased obesity-related risks
Fat percentage (%)	20.1 ± 3.7	17.0 ± 3.0	−3.1%	0.001	−4.37 to −1.83	0.92 (medium)	Moderate effect: Suggests a meaningful reduction in body fat percentage, improving body composition and possibly enhancing overall fitness levels

**Table 5:** Blood lipid measurement values according to the exercise program applied.

Variables	Pre	Post	Change (*Δ*)	*p*	95% CI	Cohen *d*	Effect size interpretation
	x̄ ± SD	x̄ ± SD					
Triglyceride (Mg/dL)	124.9 ± 11.6	116.0 ± 10.1	−8.9 mg/dL	0.001	−12.98 to −4.82	0.81 (medium)	Moderate effect: Indicates a meaningful reduction in triglyceride levels, which may contribute to improved lipid metabolism and cardiovascular health
Total cholesterol (Mg/dL)	174.4 ± 9.9	154.7 ± 6.5	−19.7 mg/dL	0.020	−22.95 to −16.45	1.98 (large)	Large effect: Suggests a substantial decrease in total cholesterol, potentially reducing the risk of atherosclerosis and cardiovascular disease
HDL (Mg/dL)	43.5 ± 3.1	53.5 ± 2.2	+10 mg/dL	0.006	8.97 to 11.03	≥2.0 (very large)	Very large effect: A strong increase in HDL, which is protective against heart disease and supports lipid metabolism
LDL (Mg/dL)	123.8 ± 3.7	115.6 ± 4.6	−8.2 mg/dL	0.004	−9.78 to −6.62	1.96 (large)	Large effect: Demonstrates a significant drop in LDL levels, reducing the risk of plaque buildup in arteries and cardiovascular complications

Recent studies highlight the efficacy of HIIT protocols in improving anthropometric characteristics, body composition, and cardiometabolic health, particularly in populations at risk for metabolic disorders. For example, research by Batrakoulis et al. (2) demonstrated that bodyweight-based HIIT programs significantly improve fat oxidation and insulin sensitivity in overweight individuals, reinforcing the present study’s findings on lipid metabolism improvements. Similarly, Batrakoulis et al. (25) reported substantial benefits of HIIT for both aerobic fitness and psychological well-being, emphasizing that shorter-duration but high-intensity exercise may be more effective than traditional endurance training for overweight populations. These findings align with the results of the current study, which demonstrated significant reductions in total cholesterol and body fat percentage following an 8-week HIIRT protocol.

Furthermore, research has underscored the importance of tailoring HIIT regimens to specific populations to optimize outcomes. Batrakoulis et al. (26) found that HIIT positively impacts neuromuscular performance and functional movement quality, even in older adults. Additionally, Batrakoulis et al. (27) suggested that incorporating individualized intensity adjustments enhances the metabolic benefits of interval training, potentially explaining the observed improvements in lipid metabolism in the present study. Future studies should explore the dose–response relationship between HIIRT intensity and metabolic adaptations, as proposed by Batrakoulis et al. (28), to establish optimal exercise prescriptions for different population groups.

Based on the results of the present study, it has been reported in the literature that lipids used as an energy source during exercise include circulating fatty acids bound to albumin, triglycerides stored in very low density lipoproteins and intramuscular triglyceride stores. In addition, dynamic changes in these lipid pools occur during and after exercise and are thought to be key factors that may regulate changes in fat oxidation in response to different exercise conditions (29).

A review of the literature reveals that studies using high-intensity resistance training (HIIRT) have reported positive effects on lipid metabolism, though results are not entirely consistent across different populations. Tuttor et al. (30) found that HIIRT improved HDL levels, resting glucose, and triglycerides, concluding that such training could be an effective strategy to reduce cardio-metabolic risk. Similarly, Da Silva et al. (31) demonstrated that a 12-week combination of resistance training (RT) and HIIT led to significant reductions in LDL levels, fasting blood glucose (FPG), and insulin resistance in both adults and older adults individuals. These findings align with the current study, which also observed favorable lipid profile changes following an 8-week HIIRT intervention.

However, discrepancies exist in the literature regarding the extent of these effects. For instance, Marcangeli et al. (32) reported that HIIT significantly improved lean mass, muscle strength, and waist circumference in obese older adults, while the current study primarily focused on lipid metabolism rather than anthropometric measures such as waist-to-hip ratio. Furthermore, some studies have failed to observe significant improvements in lipid parameters following HIIT-based training (33, 34), suggesting that individual differences in baseline fitness levels, hormonal status, dietary habits, and adherence to training protocols may influence outcomes. Additionally, gender-related differences must be considered, as Barranco-Ruiz et al. (35) highlighted that sedentary working hours disproportionately affect women, potentially impacting their metabolic responses to exercise interventions. Given these variations, further research is needed to determine the optimal HIIRT duration, intensity, and frequency for different populations, particularly for women, where data remains limited.

In a study conducted on rats (mice), which was based on the assumption that high-intensity interval training (HIIT) can effectively prevent the progression of metabolic diseases, it was found that 8 weeks of HIIT could improve lipid metabolism disorders in rats (36). Previous studies with similar exercise mode and intensity design but with longer intervention duration (4–8 months) have shown significant effects on Total cholesterol, LDL, HDL, and TG (37–40). In fact, our research also shows that the high-intensity interval training (HIIT) program enables improvements in fat metabolism. In some studies, no significant effect on blood parameters was found (33, 34, 41, 42).

The current study showed that resistance training improved body composition by reducing body weight and fat percentage in women (Table 4). The results of recent studies have shown that high-intensity resistance training is more feasible and time-efficient than moderate-intensity training and that it is effective in improving body composition, mitochondrial function, insulin sensitivity and glycemic control (43, 44). Kanaley et al. (43) found that it led to weight loss in obese individuals following high-intensity exercise. Wewege et al. (45), in their investigation of the effects of high-intensity interval training (HIIT) compared to continuous moderate-intensity training, concluded that short-term moderate to high-intensity training can lead to modest improvements in body composition in overweight and obese individuals without significant changes in body weight. In addition, it is known that after aerobic exercise, blood triglycerides decrease, total cholesterol either decreases or remains unchanged, while high-density lipoprotein (HDL) cholesterol usually increases and low-density lipoprotein (LDL) cholesterol decreases (45). In addition to studies with similar results to the current study, there are also various studies in the literature that show that high-intensity resistance exercise has no effect on body composition (21, 34, 46, 47).

These contradictory findings suggest that factors such as training duration, exercise protocol, age, gender and physical activity history of the participants may significantly affect the responses to resistance exercise. The HIIRT protocol applied in the present study contributed to positive changes in body composition by engaging both aerobic and anaerobic energy systems. In particular, the study results suggest that high intensity resistance exercise may be effective in reducing fat mass in women.

However, it should be borne in mind that individuals’ physiological responses to exercise may vary depending on genetic, hormonal and metabolic factors. In addition, differences in baseline characteristics such as younger age, sedentary status, and the use of a bodyweight-based progressive HIIRT protocol in our study may partly explain why our findings showed significant effects, whereas some previous studies using different protocols or populations did not observe similar outcomes. Therefore, future studies need to further investigate the interaction between exercise intensity, duration and individual metabolic responses and more fully evaluate the biological mechanisms that may explain these differences. In addition, long-term follow-up studies are crucial to determine the lasting effects of HIIRT on body composition and metabolic health. Moreover, HIIRT’s time-efficient structure and exercise variety may positively influence motivation and adherence, as suggested in previous literature (18, 25). In the current study, the absence of dropouts and full program completion further support its feasibility and participant engagement.

## 5 Recommendations

Given the promising outcomes observed in the present study, future research should focus on longitudinal investigations assessing the sustained impact of HIIRT on body composition and metabolic health. A key area of interest is determining the dose–response relationship of HIIRT, particularly in populations with varying baseline fitness levels. Batrakoulis et al. (48) emphasized the necessity of controlled clinical trials to evaluate how different HIIRT durations and intensities affect metabolic flexibility, insulin resistance, and lipid metabolism. Investigating the psychological effects of HIIRT, such as adherence rates and motivation levels, could also enhance its applicability in public health interventions. Moreover, future studies should consider gender-specific metabolic responses to HIIRT, as prior research has demonstrated potential sex differences in fat oxidation rates and cardiovascular adaptations to high-intensity training. It should also be acknowledged that psychological responses to HIIRT, such as perceived enjoyment, difficulty, and self-efficacy, may vary among individuals. Future studies should explore these factors to better understand their role in adherence and long-term outcomes. This training approach may also be considered as a practical strategy in public health programs or fitness interventions for sedentary women. Moreover, this type of bodyweight-based HIIRT program may serve as a practical and accessible intervention strategy for sports practice and public health initiatives aimed at improving metabolic health in sedentary women.

## 6 Conclusion

The findings of this study indicate that an 8-week High-Intensity Interval Resistance Training (HIIRT) program induces significant improvements in body composition and lipid metabolism in physically inactive women with overweight. The results demonstrated a notable reduction in body weight and fat percentage, as well as positive alterations in lipid profiles, including a decrease in triglyceride, total cholesterol, and LDL levels and a substantial increase in HDL levels. These findings suggest that HIIRT is an effective and time-efficient training modality for enhancing cardiovascular and metabolic health in physically inactive populations.

## 7 Limitations

This study has several limitations that should be acknowledged. One limitation of the present study is the lack of a control group. A single-group pre-post design was chosen due to the exploratory nature of the study and logistical constraints related to participant recruitment and scheduling. Future research should include randomized controlled trials to further validate these findings.

First, although bioelectrical impedance analysis (BIA) was used to assess body composition due to its practicality and accessibility, more precise techniques such as Dual-Energy X-ray Absorptiometry (DEXA) could have provided a more detailed evaluation of fat distribution, lean mass, and visceral adiposity. Future studies should incorporate DEXA scanning or other advanced imaging techniques to enhance the accuracy of body composition assessments.

Second, this study did not include anthropometric measurements such as waist circumference and waist-to-hip ratio, which are crucial indicators of central adiposity and cardiometabolic risk. Given that abdominal fat accumulation is closely linked to metabolic syndrome and cardiovascular diseases, future research should integrate these measures to provide a more comprehensive analysis of training effects on fat distribution and metabolic health.

Third, the study lacked a control group, which limits the ability to determine whether the observed improvements in body composition and lipid metabolism were solely due to the HIIRT intervention or if other external factors (e.g., lifestyle changes, dietary modifications, or seasonal variations) may have influenced the results. Future research should employ randomized controlled trial (RCT) designs to establish stronger causal relationships between HIIRT and metabolic health outcomes.

Additionally, the study’s sample size was relatively small (*n* = 30) and included only sedentary young women, which may limit the generalizability of the findings to other populations, such as men, older adults, or individuals with metabolic disorders. Expanding the sample size and including more diverse participant groups could provide a broader understanding of HIIRT’s effects across different populations.

Finally, the short intervention period (8 weeks) restricts the ability to assess long-term physiological adaptations and sustainability of improvements in body composition and metabolic parameters. While significant positive changes were observed within this timeframe, longitudinal studies are needed to determine whether HIIRT-induced benefits persist over time and to evaluate the potential for weight regain or metabolic reversals after training cessation.

## 8 Practical implications

The findings of this study highlight the potential of HIIRT as a time-efficient and accessible exercise strategy for improving body composition and lipid metabolism in sedentary women. Given its effectiveness in reducing body fat, LDL, and triglycerides while increasing HDL, HIIRT can be incorporated into clinical exercise prescriptions for managing obesity and metabolic disorders. Healthcare professionals may recommend HIIRT as a non-pharmacological intervention to reduce cardiovascular risk, while fitness trainers can implement structured HIIRT programs to enhance fat loss and functional strength in clients with limited access to gym facilities. Additionally, public health initiatives could integrate HIIRT into community-based programs to promote physical activity and metabolic health in inactive populations.

Future research should focus on optimizing HIIRT protocols for different age groups and fitness levels to enhance its real-world applicability.

## Data Availability

The raw data supporting the conclusions of this article will be made available by the authors, without undue reservation.
